# Pan-cancer analysis of CLDN18.2 shed new insights on the targeted therapy of upper gastrointestinal tract cancers

**DOI:** 10.3389/fphar.2024.1494131

**Published:** 2024-11-01

**Authors:** Jun Wu, Jinghua Lu, Qiuyue Chen, Haojie Chen, Yongqiang Zheng, Minggang Cheng

**Affiliations:** ^1^ Department of Clinical Laboratory, People's Hospital of Bao'an District, Shenzhen Baoan Clinical Medical College of Guangdong Medical University, Shenzhen, China; ^2^ State Key Laboratory of Oncology in South China, Guangdong Provincial Clinical Research Center for Cancer, Sun Yat-sen University Cancer Center, Guangzhou, China

**Keywords:** CLDN18.2, alternative splicing events, upper gastrointestinal tract cancers, prognosis, drug sensitivity

## Abstract

**Background:**

CLDN18.2 is a widely researched drug target. However, previous research has primarily been based on immunohistochemistry results and focused on gastric cancer.

**Methods:**

To analyze the potential cancer-targeting effect of CLDN18.2 from a multi-omics perspective, this study quantified CLDN18.2 expression in The Cancer Genome Atlas (TCGA) pan-cancer cohort. Thus, the relationships between CLDN18.2 expression and genomic alterations, immune infiltration, and prognosis were analyzed. Additionally, we performed analyses of the differentially expressed genes and enriched pathways between the high- and low-CLDN18.2 expression groups, as well as the corresponding drug sensitivity analyses.

**Results:**

The results indicated that CLDN18.2 was highly expressed in pancreatic adenocarcinoma (PAAD), stomach adenocarcinoma (STAD), colorectal cancer (CRC), and esophageal carcinoma (ESCA). Moreover, the high- and low-CLDN18.2 expression groups presented significant differences in terms of genomic alterations and immune infiltration, such as the levels of methylation and CD4^+^ T cell infiltration. Furthermore, high CLDN18.2 expression was significantly associated with poor prognosis in bladder urothelial carcinoma (BLCA), ESCA, and PAAD. In upper gastrointestinal tract cancers (STAD, ESCA, and PAAD), downregulated gene-enriched pathways were associated with cell signaling, whereas upregulated gene-enriched pathways were associated with angiogenesis. Finally, we identified drugs associated with CLDN18.2 expression to which samples with different levels of expression were differentially sensitive.

**Conclusion:**

CLDN18.2 was highly expressed in upper gastrointestinal tract cancers, and its expression had a significant effect on genomic alterations and the tumor microenvironment. Additionally, low CLDN18.2 expression was linked to favorable prognosis. Our study reveals the potential value of CLDN18.2 for tumor prognosis and targeted therapy in various cancers, especially upper gastrointestinal tract cancers.

## 1 Introduction

In terms of current global disease trends, cancer remains one of the leading causes of death among non-communicable diseases and poses an enormous burden on social development and healthcare resources (Vollset et al., 2024). According to a statistical analysis of data from the International Agency for Research on Cancer (IARC) in 2022, lung, breast, colorectal, prostate, and gastric cancers are at the forefront of morbidity and mortality, which cause irreversible physical and financial losses to a large number of patients and their families (Bray et al., 2024; Jokhadze et al., 2024). Currently, seven primary treatments are available for tumors, namely, surgery (Are et al., 2023), radiotherapy (Schaue and McBride, 2015), chemotherapy (Anand et al., 2023), immunotherapy (Carlino et al., 2021), targeted therapy (Zhong et al., 2021), hormone therapy (Yung and Davidson, 2021) and stem cell transplantation (Chu et al., 2020). Among them, targeted therapy is a type of specific therapy that inhibits specific molecular targets of cancer cells to hinder their growth and proliferation, which has already played an important role in the treatment of multiple cancers, such as lung (Niu et al., 2022), breast (Esteva et al., 2019), and gastric (Zhu et al., 2021) cancers.

The claudin18 gene (CLDN18) is a coding gene belonging to the claudin family that is often expressed in epithelial cells, where it plays an important role in intercellular junctions and maintenance of cell polarity (Günzel and Yu, 2013). The human CLDN18 gene is located on chromosome 3 (3q22.3) and contains six exons and five introns, as well as two alternate promoters (APs). The two promoters mediate different transcription start sites, which in turn affect different downstream exons (1a and 1b), resulting in two isoforms, CLDN18.1 and CLDN18.2 (Niimi et al., 2001). The structures of the two isoforms are very similar, with four hydrophobic transmembrane structural domains and two extracellular loops, and they differ in only a few amino acids at the N-terminal first extracellular loop, which leads to differences in their functions and expression specificity (Türeci et al., 2011). CLDN18.1 is expressed primarily in the lungs, whereas CLDN18.2 is expressed predominantly in the stomach (Niimi et al., 2001). In gastric cancer, CLDN18.2 is predominantly located in the apical tight junctions of epithelial cells in normal tissues, whereas it is exposed in tumor cells due to alterations in the junctions and polarity between epithelial cells as a result of epithelial mesenchymal transition (EMT) and is also ectopically activated in tumor tissues from various other cancers (Sahin et al., 2008).

Therefore, CLDN18.2 has the potential to be a cancer therapeutic target (Nakayama et al., 2024). Currently, CLDN18.2 is a widely researched target for cancer therapy, and a series of therapeutic agents have been developed. Zolbetuximab, the first monoclonal antibody to target CLDN18.2, can activate immune cells through the antibody-dependent cellular cytotoxicity (ADCC) mechanism and activate the complement system through the complement-dependent cytotoxicity (CDC) mechanism, both of which synergistically achieve therapeutic effects in cells that specifically express CLDN18.2 (Mitnacht-Kraus et al., 2017). In addition to monoclonal antibodies, other targeted therapies based on CLDN18.2, such as chimeric antigen receptor T cells (CAR T cells), bispecific antibodies, and antibody-drug conjugates (ADCs), have been developed. These therapies have made notable progress in the targeted treatment of cancers such as gastric, esophageal, and pancreatic cancers (Jiang et al., 2019; Shah et al., 2023; Shitara et al., 2023; Xu R.-h. et al., 2023; Xu Y. et al., 2023).

Moreover, several studies have analyzed the pathological characteristics and prognosis of CLDN18.2-positive patients. Wang et al. found that gastric cancer tumor tissues exhibited lower levels of CLDN18.2 expression compared to normal tissues. Furthermore, the combination of low CLDN18.2 expression and increased infiltration of CD4^+^ or CD8^+^ T cells was associated with a better prognosis (Wang et al., 2023). Kwak et al. analyzed the association between CLDN18.2 and several biomarkers in gastric cancer. This study demonstrated that the positive rate of CLDN18.2 was higher in patients with EBV-positive or PD-L1-positive gastric cancer, whereas it was lower in HER2-positive patients (Kwak et al., 2024). However, related research primarily uses immunohistochemistry to determine CLDN18.2 expression, focusing primarily on gastric cancer and lacking a multi-omics landscape of CLDN18.2 across various cancers. Additionally, current transcript sequencing technologies have focused on quantifying the expression of each gene rather than each isoform (Garber et al., 2011; Stark et al., 2019), and the data from numerous bioinformatics databases rarely involve specific isoforms, which makes analyzing the biological properties of CLDN18.2 from a multi-omics perspective difficult. The TCGASpliceSeq database (https://bioinformatics.mdanderson.org/TCGASpliceSeq) utilizes the data associated with The Cancer Genome Atlas (TCGA) project to provide a comprehensive and detailed interpretation of the alternative splicing patterns of these samples and quantify the corresponding percent spliced in (PSI) values for splice events (Ryan et al., 2016). This pioneering work presents possibilities for us to quantify and subsequently analyze CLDN18.2 at the transcriptome level.

**FIGURE 1:**
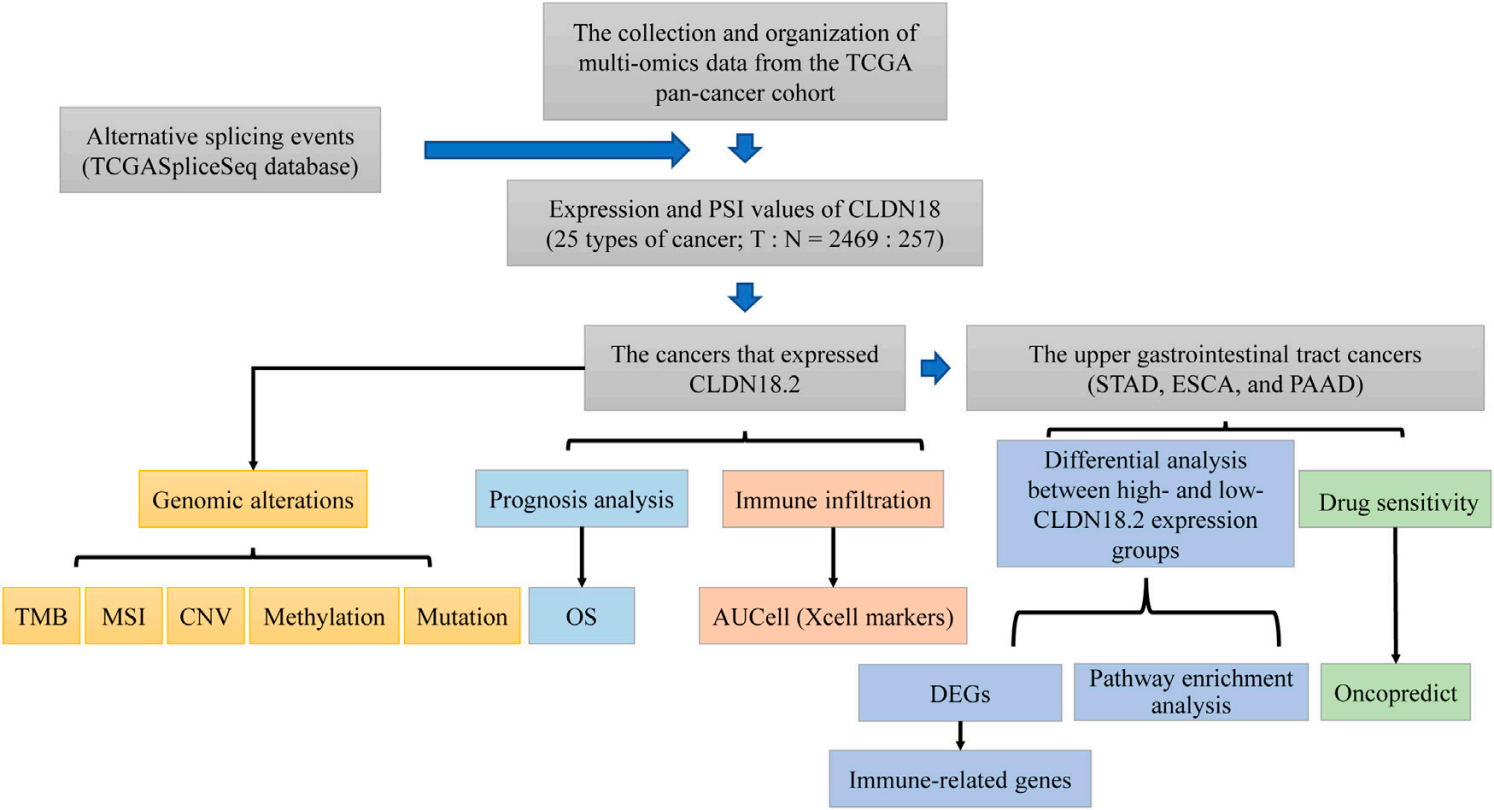
The workflow of this study.

Therefore, this study analyzed CLDN18.2 expression across cancers and the associations of CLDN18.2 expression with genomic alterations, immune infiltration, and prognosis from a multi-omics perspective. In upper gastrointestinal tract cancers with high CLDN18.2 expression, we further analyzed the differences in gene expression, enriched pathways, and drug sensitivity between the high- and low-expression groups. The workflow of this study was shown in Figure 1. Taken together, our findings expand the current state of research on CLDN18.2 across various cancers, especially upper gastrointestinal tract cancers, and provide new insights for subsequent CLDN18.2-based targeted therapy.

## 2 Materials and methods

### 2.1 Data collection and preprocessing

Alternative splicing data for CLDN18 were obtained from the TCGASpliceSeq database, and all cancer data containing PSI values for CLDN18 were downloaded (26 cancers). Multi-omics data of the pan-cancer cohort in TCGA, including RNA-seq data, clinical information, methylation data, and copy number variant (CNV) data, were downloaded from the UCSC Xena (Goldman et al., 2020) database (https://xenabrowser.net/datapages/). Tumor mutation burden (TMB) and microsatellite instability (MSI) data were obtained from the cBioPortal (Cerami et al., 2012) database (https://www.cbioportal.org/), whereas tumor neoantigen data was sourced from the Supplementary Material in the literature on the pan-cancer immune landscape of TCGA (Thorsson et al., 2018). Moreover, we downloaded the mutation data via the “TCGAMutations” (v0.4.0) R package. The above data were filtered to retain only those samples that included both the PSI values of CLDN18 and its expression profiles. In addition, samples from colon adenocarcinoma (COAD) and rectum adenocarcinoma (READ) were mixed together and defined as colorectal cancer (CRC).

### 2.2 Expression of CLDN18.2 and CLDN18.1

For RNA-seq data, fragments per kilobase million (FPKM) values were converted to transcripts per million (TPM) values, and then the “clusterProfiler” (v4.10.1) R package (Wu et al., 2021) was used to convert Ensembl IDs to gene symbols while filtering out unmatched records. Building upon this, we partitioned CLDN18 expression into CLDN18.2 and CLDN18.1 based on the proportion of AP, where AP1 corresponds to CLDN18.2 and AP2 corresponds to CLDN18.1, totaling 1 (Supplementary Table S1). The samples from each cancer type were categorized into high- and low-expression groups according to the median expression value of CLDN18.2.

We demonstrated the expression of CLDN18, CLDN18.1, and CLDN18.2 among cancers and between tumor samples and normal samples. Additionally, PSI values were presented for each type of cancer and for comparisons between tumor samples and normal samples. We visualized the information about cancer samples based on the sorting of PSI values for CLDN18.2. Differences between the above data were analyzed with the Kruskal–Wallis test and the Wilcoxon rank sum test and visualized by the “ggplot2” (v3.5.1) and “complexHeatmap” (v2.18.0) R packages.

### 2.3 Genomic alterations

In our study, ten cancers expressed CLDN18.2, namely, bladder urothelial carcinoma (BLCA), cholangiocarcinoma (CHOL), CRC, esophageal carcinoma (ESCA), liver hepatocellular carcinoma (LIHC), lung adenocarcinoma (LUAD), lung squamous cell carcinoma (LUSC), pancreatic adenocarcinoma (PAAD), stomach adenocarcinoma (STAD), and testicular germ cell tumors (TGCT). Thus, we analyzed the correlation between CLDN18.2 expression and TMB or MSI in these cancers. Moreover, differences in the levels of methylation and CNV between the high- and low-CLDN18.2 expression groups across these cancers were analyzed via the Wilcoxon rank sum test. In four cancers with high CLDN18.2 expression (STAD, CRC, ESCA, and PAAD), we explored the differences in mutated genes between the high- and low-CLDN18.2 expression groups using the “maftools” (v2.18.0) R package.

### 2.4 Immune infiltration analysis

For the cancers that expressed CLDN18.2, we calculated the level of immune cell infiltration in each sample using the “AUCell” (v1.24.0) (Aibar et al., 2017) R package, which is based on Xcell (Aran, 2020) markers. We subsequently assessed the correlation between CLDN18.2 expression and the infiltration level of each immune cell type using the “cor.test” R function and visualized it using the “complexHeatmap” R package. For the four cancers with high CLDN18.2 expression, the immune cell infiltration levels of the high- and low-CLDN18.2 expression groups were compared, and the differences between the two groups were analyzed with the Wilcoxon rank sum test.

### 2.5 Prognosis analysis

For the cancers that expressed CLDN18.2, after the optimal cutoff point was determined by the “surv_cutpoint” function of the “survminer” (v0.4.9) R package, we analyzed the relationship between CLDN18.2 expression and overall survival (OS) across these cancers using the “survival” (v3.6-4) R package and visualized them with Kaplan–Meier plots. Furthermore, the corresponding log-rank P values and hazard ratios (HRs) with 95% confidence intervals were visualized by the “forestplot” (v3.1.3) R package. In addition, as patients’ HER2 status is a crucial consideration in gastric cancer treatment, we identified HER2-amplified samples in STAD using CNV data, where a CNV score of 1 indicates amplification. We then analyzed the correlation between CLDN18.2 expression and ERBB2 expression in STAD, as well as the association between CLDN18.2 expression and OS in HER2-amplified samples.

### 2.6 Differential gene expression and enrichment analysis between the high- and low-CLDN18.2 expression groups

In this study, CLDN18.2 was highly expressed in four cancers (STAD, CRC, ESCA, and PAAD), but high CLDN18.2 expression may improve the prognosis of patients with CRC, which was different from the results of the other three cancers. Therefore, subsequent analysis focused on the remaining three cancers. Because the pancreas and upper gastrointestinal tract are anatomically proximate, we classified pancreatic cancer as upper gastrointestinal tract cancer for follow-up analysis.

In upper gastrointestinal tract cancers (STAD, ESCA, and PAAD), we explored the differentially expressed genes (DEGs) between the high- and low-CLDN18.2 expression groups using the “DESeq2” (v1.42.1) R package, where genes with the false discovery rate (FDR) < 0.05 and a |fold change| > 2 were considered significant.

The overlaps of differential gene results for the three cancers were calculated and visualized by the “ggvenn” (v0.1.10) R package. Moreover, we performed Kyoto Encyclopedia of Genes and Genomes (KEGG) pathway and hallmark pathway enrichment analyses on the overlapping differential gene sets from the three cancers and visualized them using the “clusterProfiler” R package. Additionally, we used the “ggplot2” and “complexHeatmap” R packages to visualize the expression of immune-related genes between the high- and low-CLDN18.2 expression groups and their correlation with CLDN18.2 expression.

### 2.7 Drug sensitivity analysis

The Cancer Therapeutics Response Portal (CTRP) database (https://portals.broadinstitute.org/ctrp.v2.1/) collects a large amount of data on interactions between cell lines and related compounds. And it is designed to assist in identifying drugs beneficial to patients by correlating the cellular characteristics (genetic, lineage, etc.) of cancer cell lines with their sensitivity to small molecules (Rees et al., 2016). Oncopredict (v1.2) is an R package designed to be used for drug response prediction and drug–gene association prediction based on data from the CTRP database, which predicts the drug sensitivity of samples to 545 drugs (Maeser et al., 2021). We utilized this package to predict the sensitivity of samples from the upper gastrointestinal tract cancers to 545 drugs. We then visualized the results of the sensitivity score ranking and several drugs with the highest correlation with CLDN18.2 expression using the “ggplot2” R package. To explore the differences in drug sensitivity scores between the high- and low-CLDN18.2 expression groups, we selected the top 100 drugs with the lowest scores in each cancer and calculated their correlation with CLDN18.2 expression. Drugs with correlations greater than 0.4 and significant differences between the high- and low-CLDN18.2 expression groups were then displayed.

### 2.8 Statistical analysis

In this study, we compared differences between two groups and between multiple groups using the Wilcoxon rank sum test and the Kruskal–Wallis test, respectively. The data correlation was calculated via Pearson’s correlation. All the above methods were performed with R (v4.3.1) software.

## 3 Results

### 3.1 Expression of CLDN18, CLDN18.2 and CLDN18.1 across cancers

**FIGURE 2:**
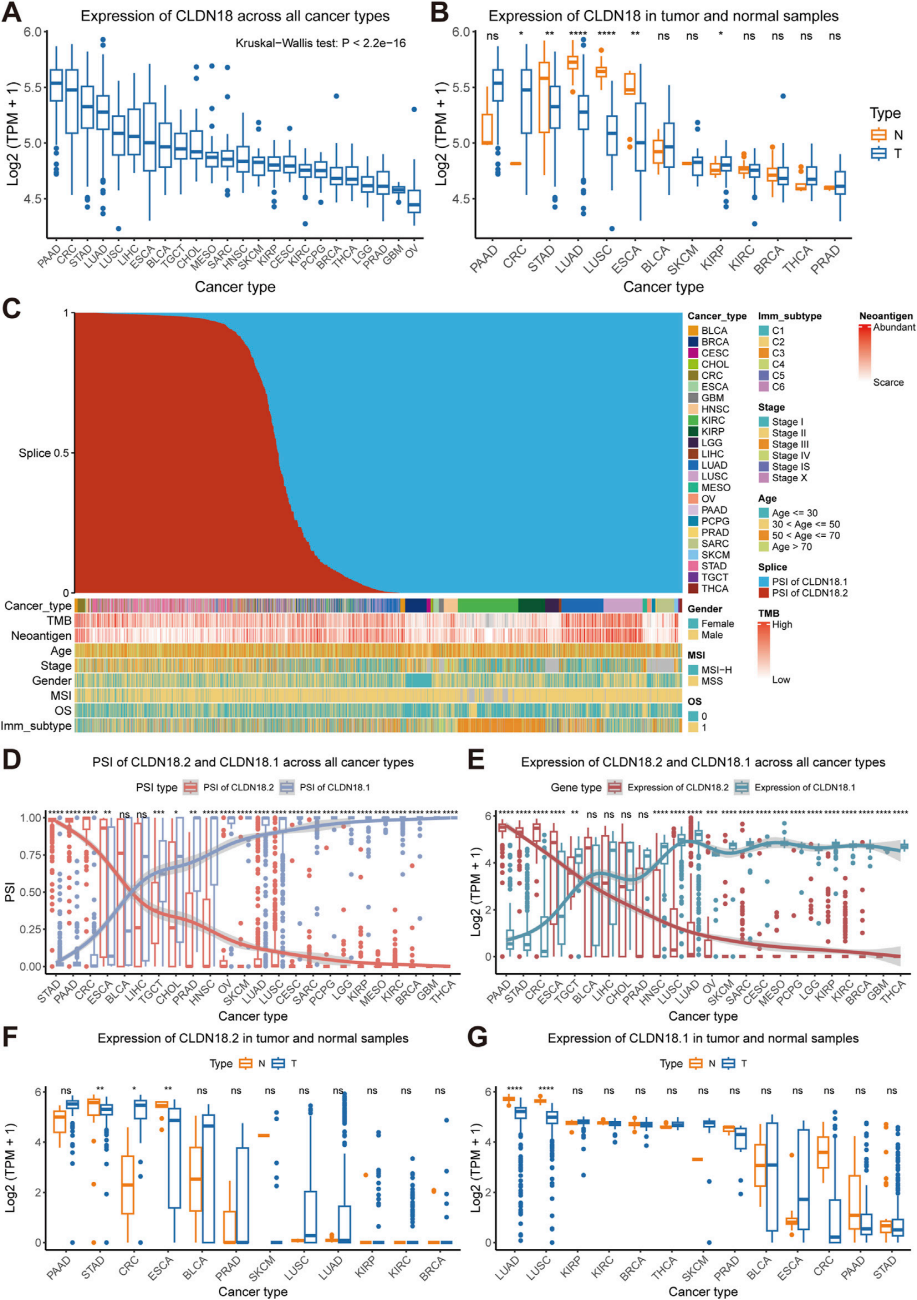
Expression and alternative splicing events of CLDN18, CLDN18.2, and CLDN18.1. **(A)** CLDN18 expression across various cancers. **(B)** CLDN18 expression in tumor and normal samples across various cancers. **(C)** Details of cancer samples after sorting by PSI values. **(D)** PSI values of CLDN18.2 and CLDN18.1 across various cancers. **(E)** Expression of CLDN18.2 and CLDN18.1 across various cancers. The expression of CLDN18.2 **(F)** and CLDN18.1 **(G)** in tumor and normal samples across various cancers. *, P < 0.05; **, P < 0.01; ***, P < 0.001; ****, P < 0.0001; ns, not statistically significant. Note: BLCA, Bladder urothelial carcinoma; BRCA, Breast invasive carcinoma; CESC, Cervical squamous cell carcinoma and endocervical adenocarcinoma; CHOL, Cholangiocarcinoma; CRC, Colorectal cancer; ESCA, Esophageal carcinoma; GBM, Glioblastoma multiforme; HNSC, Head and neck squamous cell carcinoma; KICH, Kidney chromophobe; KIRC, Kidney renal clear cell carcinoma; KIRP, Kidney renal papillary cell carcinoma; LGG, Lower grade glioma; LIHC, Liver hepatocellular carcinoma; LUAD, Lung adenocarcinoma; LUSC, Lung squamous cell carcinoma; MESO, Mesothelioma; OV, Ovarian serous cystadenocarcinoma; PAAD, Pancreatic adenocarcinoma; PCPG, Pheochromocytoma and paraganglioma; PRAD, Prostate adenocarcinoma; SARC, Sarcoma; SKCM, Skin cutaneous melanoma; STAD, Stomach adenocarcinoma; TGCT, Testicular germ cell tumors; THCA, Thyroid carcinoma.

In the TCGA pan-cancer cohort, PAAD, CRC, STAD, and LUAD expressed higher levels of CLDN18, whereas lower grade glioma (LGG), prostate adenocarcinoma (PRAD), glioblastoma multiforme (GBM), and ovarian serous cystadenocarcinoma (OV) had lower levels of CLDN18 expression (Figure 2A). Further comparison between tumor samples and normal samples revealed significant differences in CRC, STAD, LUAD, LUSC, ESCA, and kidney renal papillary cell carcinoma (KIRP), and the CLDN18 expression levels in the remaining four cancers were significantly higher in normal samples than in tumor samples, except for CRC and KIRP (Figure 2B).

Sorting the PSI values of CLDN18 revealed that the distribution of cancer types in samples with higher PSI values of CLDN18.2 was more diverse, but the distribution of cancer types in samples with higher PSI values of CLDN18.1 was more concentrated and included breast invasive carcinoma (BRCA), kidney renal clear cell carcinoma (KIRC), KIRP, LUAD, LUSC, and sarcoma (SARC) (Figure 2C). We compared the PSI values of CLDN18.2 and CLDN18.1, and the results showed no significant difference only in BLCA and LIHC (Figure 2D). In addition, there were no significant differences in the PSI values of two isoforms between tumor samples and normal samples across other cancers except for CRC (Supplementary Figure S1).

Further analysis of the differences in expression between CLDN18.2 and CLDN18.1 revealed significant differences in results for all cancer types except for BLCA, LIHC, CHOL, and PRAD (Figure 2E). Moreover, CLDN18.2 expression significantly differed between tumor samples and normal samples in patients with STAD, CRC, and ESCA (Figure 2F). Correspondingly, CLDN18.1 expression between tumor samples and normal samples was significantly different only in LUAD and LUSC (Figure 2G). These results indicate highly specific expression levels of CLDN18.2 and CLDN18.1 across different cancers, with higher CLDN18.2 expression in PAAD, STAD, CRC, and ESCA and higher CLDN18.1 expression in LUAD and LUSC.

### 3.2 Genomic alterations in cancers that expressed CLDN18.2

**FIGURE 3:**
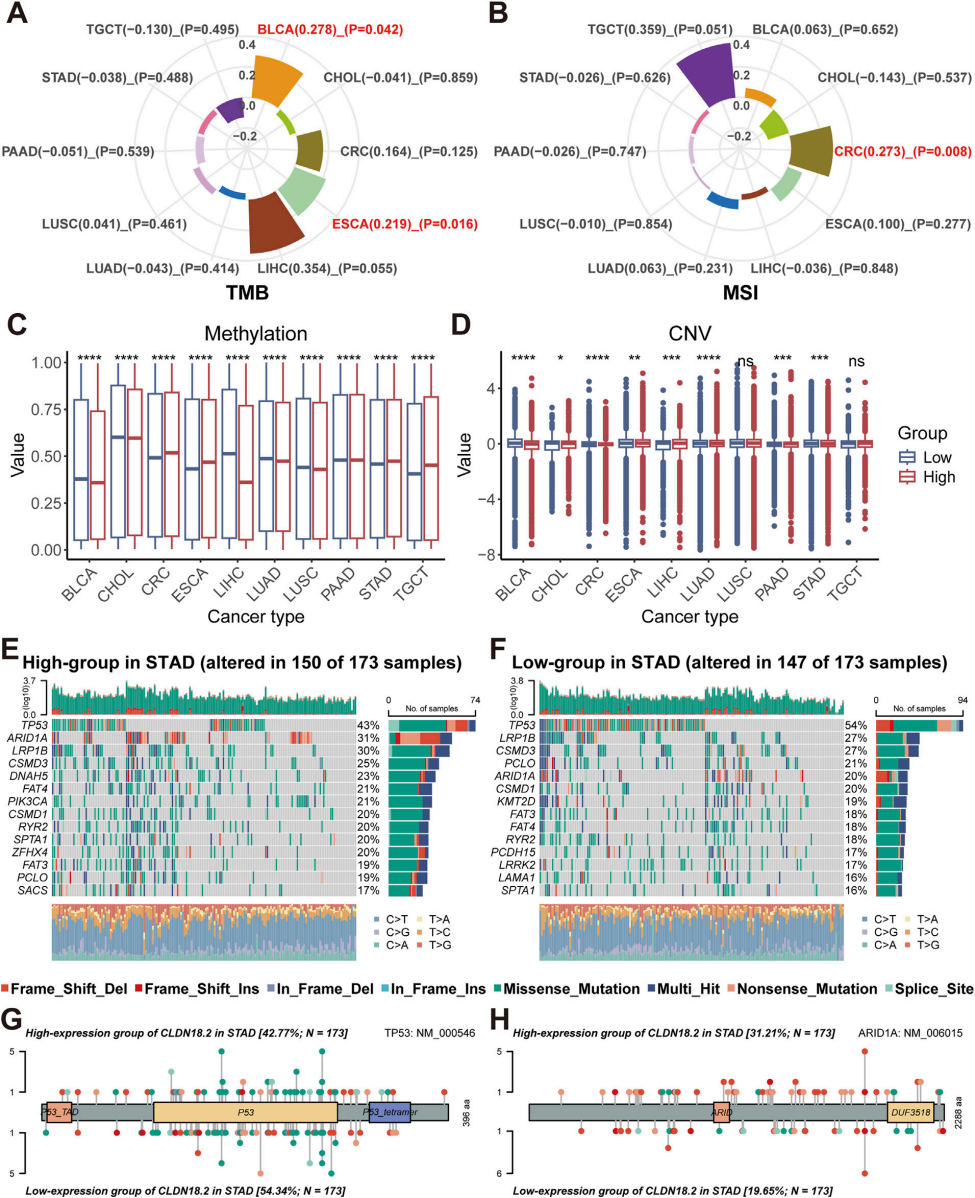
Relationships between CLDN18.2 expression and genomic alterations across ten cancers. Correlations of TMB **(A)** and MSI **(B)** with CLDN18.2 expression across ten cancers. Differences in levels of methylation **(C)** and CNV **(D)** between high- and low-CLDN18.2 expression groups. Mutation profiles between the high- **(E)** and low-CLDN18.2 **(F)** expression groups in STAD. Protein mutation lollipop plots for TP53 **(G)** and ARID1A **(H)** between high- and low-CLDN18.2 expression groups in STAD. *, P < 0.05; **, P < 0.01; ***, P < 0.001; ****, P < 0.0001; ns, not statistically significant.

We calculated the correlation of TMB and MSI with CLDN18.2 expression in ten cancers that expressed CLDN18.2 separately (BLCA, CHOL, CRC, ESCA, LIHC, LUAD, LUSC, PAAD, STAD, and TGCT). Although significant positive correlations were observed in BLCA (P = 0.042) and ESCA (P = 0.016), TMB and CLDN18.2 expression did not significantly correlate in other cancers (Figure 3A). For MSI, only CRC (P = 0.008) showed a significant positive correlation (Figure 3B). Moreover, the differences in methylation and CNV levels between the high- and low-CLDN18.2 expression groups were compared in each cancer type. The results indicated that methylation levels significantly differed between the high- and low-CLDN18.2 expression groups in all cancers (Figure 3C), whereas CNV levels did not significantly differ between two groups in LUSC and TGCT (Figure 3D).

For the four cancers (PAAD, STAD, CRC, and ESCA) with higher CLDN18.2 expression levels than the other cancers shown in Figure 2E, we analyzed the mutation profiles of the high- and low-CLDN18.2 expression groups. In STAD, the mutated gene types and frequencies differed between the high- and low-CLDN18.2 expression groups and were dominated by missense mutations and multiple mutations (Figures 3E, F). A comparison of the top 20 mutated genes (excluding several large genes) revealed DNAH5, PIK3CA, ZFHX4, SACS, KMT2D, PCDH15, LRRK2, and LAMA1 as the differently mutated genes between the high- and low-CLDN18.2 expression groups (Figures 3E, F). We further compared the mutations in TP53 and ARID1A and observed that the mutation rate of TP53 in the high-CLDN18.2 expression group was lower than that in the low-CLDN18.2 expression group, but the opposite was true for ARID1A (Figures 3G, H).

In the high-CLDN18.2 expression group of CRC, TP53 was not among the top-ranked mutated genes, and the APC gene mutation rate in the low-CLDN18.2 expression group was much higher than that in the high-CLDN18.2 expression group (Supplementary Figures S2A, B). The mutation profile of TP53 in ESCA was similar to that in STAD, which also indicated a higher mutation rate of TP53 in the low-CLDN18.2 expression group. In addition, among the genes with the highest mutation rate in ESCA, the overall mutation rate of the top-ranked mutated genes in the high-CLDN18.2 expression group was higher than that in the low-CLDN18.2 expression group (Supplementary Figures S2C, D). For the high- and low-CLDN18.2 expression groups of PAAD, the mutation rates of the other genes except for KRAS, TP53, SMAD4, and CDKN2A were lower. The mutation rates of KRAS and TP53 were higher in the high-CLDN18.2 expression group than in the low-CLDN18.2 expression group (Supplementary Figures S2E, F).

CLDN18 had few mutations in the four cancers, with only a small number of mutations in STAD and CRC, which predominantly consisted of missense mutations and nonsense mutations (Supplementary Figures S2G, H). Overall, the types of genomic alterations differ between the high- and low-CLDN18.2 expression groups across these cancers, especially the patterns of mutated genes.

### 3.3 Relationships between CLDN18.2 expression and immune infiltration

**FIGURE 4:**
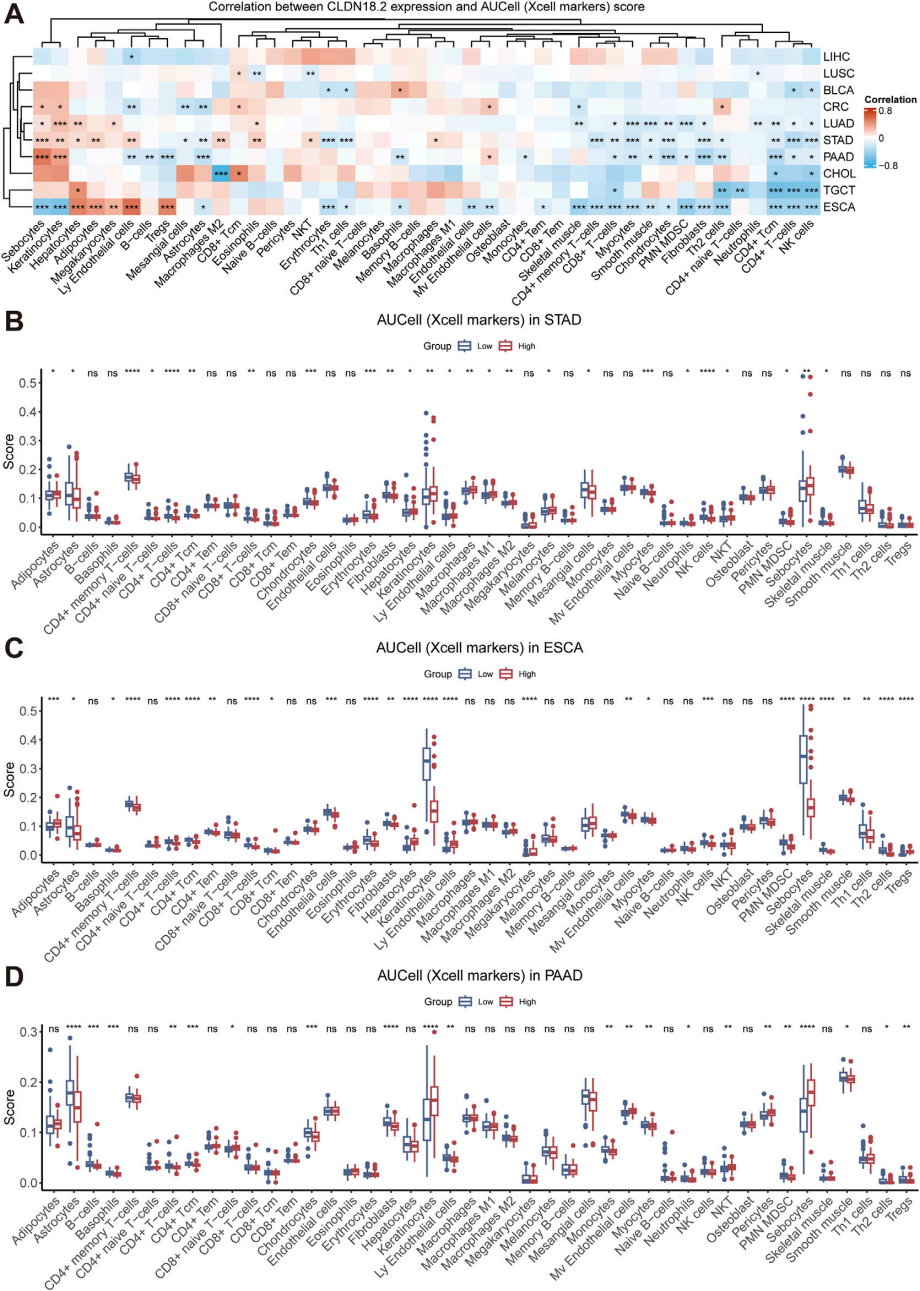
Immune infiltration analyses across ten cancers. **(A)** The correlations between CLDN18.2 expression and immune infiltration across ten cancers. **(B–D)** The levels of immune cell infiltration between high- and low-CLDN18.2 expression groups in STAD, ESCA, and PAAD. *, P < 0.05; **, P < 0.01; ***, P < 0.001; ****, P < 0.0001; ns, not statistically significant.

Tumor immune cells play a bidirectional role in cancer development, either inhibiting tumor growth by eliminating tumor cells or promoting tumor progression via immune escape mechanisms. To investigate the relationship between CLDN18.2 expression and the level of immune cell infiltration, we calculated the correlation between CLDN18.2 expression and infiltrations of various immune cells in each cancer that expressed CLDN18.2. Overall, the infiltration levels of CD4^+^ Tcm cells, CD4^+^ T cells, and NK cells and neutrophils were negatively correlated with CLDN18.2 expression in most cancers, whereas the results for other immune cells were cancer-specific (Figure 4A).

In the four cancers that expressed more CLDN18.2 than other cancers, CLDN18.2 expression was negatively correlated with CD8^+^ T cells, myocytes, chondrocytes, and fibroblasts, but this negative correlation was not significant in CRC. In STAD, CLDN18.2 expression was significantly positively correlated with M2 macrophages, eosinophils, and NKT cells and significantly negatively correlated with CD4^+^ memory T cells, CD8^+^ T cells, and Th2 cells. In PAAD, the analysis revealed a strong positive correlation between CLDN18.2 expression and both B cells and Tregs, which was not observed in the other cancers. In ESCA, CLDN18.2 expression significantly and negatively correlated with the infiltration levels of sebocytes and keratinocytes (Figure 4A).

Moreover, we analyzed the differences in the levels of immune cell infiltration between the high- and low-CLDN18.2 expression groups in patients with STAD, which identified significant differences in various immune cells, such as CD8^+^ T cells, CD4^+^ memory T cells, CD4^+^ naive T cells, CD4^+^ T cells, and CD4^+^ Tcm cells. Additionally, the high-CLDN18.2 expression group exhibited a higher level of immune infiltration in M2 macrophages compared to the low-CLDN18.2 expression group, whereas the level of NK cells infiltration was lower in the high-CLDN18.2 expression group (Figure 4B). Further combining the results of ESCA and PAAD revealed that the infiltration patterns of the CD4^+^ T cell subsets were similar between the high- and low-CLDN18.2 expression groups across the three cancers, as were those of CD8^+^ T cell subsets. However, there were significant differences in infiltration levels of macrophage subsets between the two groups in STAD, which were not observed in ESCA or PAAD (Figures 4C, D). Additionally, the infiltration level of most immune cells did not differ between the two groups in CRC (Supplementary Figure S3). These findings indicate that the effect of CLDN18.2 expression on the infiltration levels of immune cells is highly cancer-specific, and high and low CLDN18.2 expression may have differential effects.

### 3.4 Relationships between CLDN18.2 expression and OS

**FIGURE 5:**
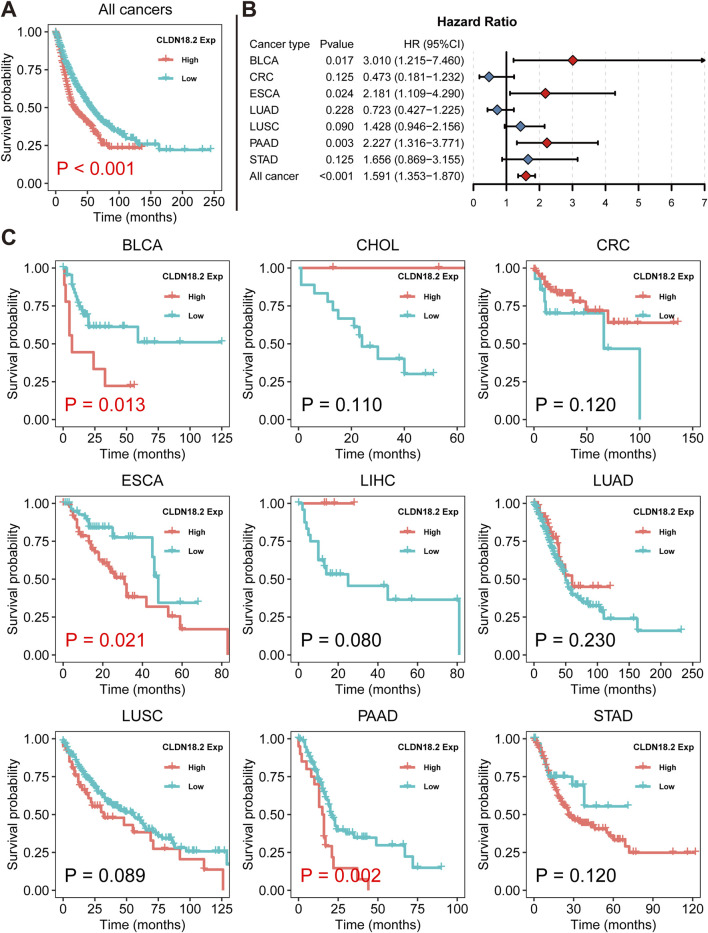
Relationships between CLDN18.2 expression and overall survival across ten cancers. **(A)** Kaplan–Meier analysis of the relationship between CLDN18.2 expression and OS across ten cancers. **(B)** Forest plot of the relationships between CLDN18.2 expression and OS across ten cancers. **(C)** Kaplan–Meier analyses of the relationship between CLDN18.2 expression and OS for individual cancers. Note: The analyses of CHOL and LIHC were not presented due to HR being very close to 0 and P values being approaching 1. And the analysis of TCGT was not presented because all samples had reached the OS endpoint.

We revealed the association between overall CLDN18.2 expression and OS across ten cancers via Kaplan–Meier analysis. Overall, low CLDN18.2 expression was associated with longer OS (P = 0.001) (Figure 5A). A Cox proportional hazards regression model showed that CLDN18.2 expression was a significant low-risk factor in BLCA patients (P = 0.017), ESCA patients (P = 0.024), PAAD patients (P = 0.003), and overall patients (P = 0.001) (Figure 5B). Moreover, Kaplan–Meier analysis of these cancers individually revealed that high CLDN18.2 expression was a poor prognostic factor in BLCA (P = 0.013), ESCA (P = 0.021) and PAAD (P = 0.002) (Figure 5C). These results indicate that CLDN18.2 expression has a guiding significance for the prognosis of several cancers, notably upper gastrointestinal tract cancers.

Through a more detailed analysis of the association between CLDN18.2 and HER2 in STAD, we found that CLDN18.2 expression was significantly positively correlated with ERBB2 expression (P = 0.023) (Supplementary Figure S4A). Furthermore, we observed that low CLDN18.2 expression was significantly associated with longer OS in HER2-amplified samples (Supplementary Figure S4B).

### 3.5 Analyses of DEGs and pathway enrichment between the high- and low-CLDN18.2 expression groups

**FIGURE 6:**
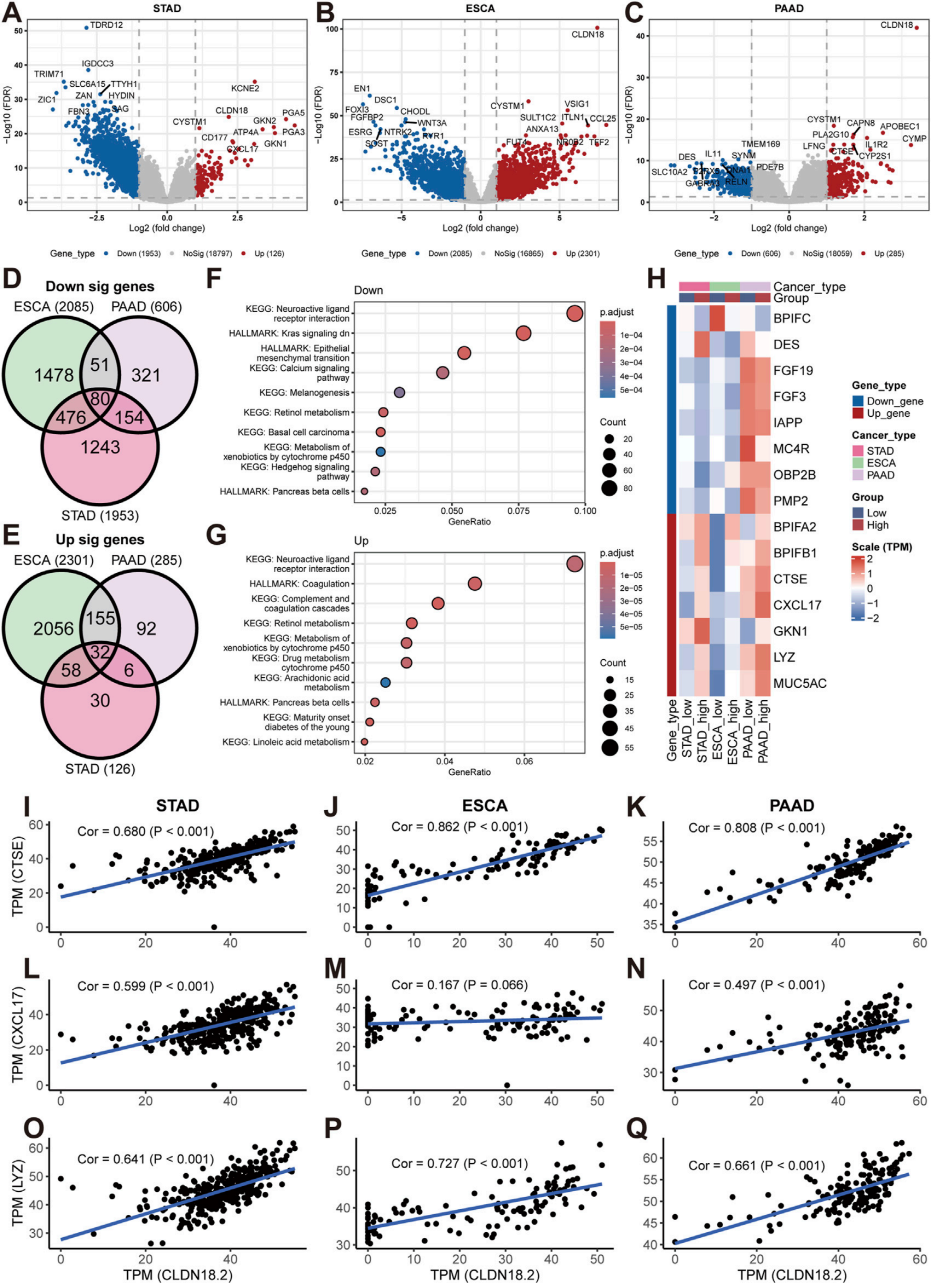
Differential analyses between high- and low-CLDN18.2 expression groups in STAD, ESCA, and PAAD. Volcano plots between high- and low-CLDN18.2 expression groups in STAD **(A)**, ESCA **(B)**, and PAAD **(C)**. Venn diagrams of upregulated genes **(D)** and downregulated **(E)** genes across three cancers. **(F, G)** KEGG and hallmark pathway enrichment plots across three cancers. **(H)** Correlations between CLDN18.2 expression and immune-related genes between high- and low-CLDN18.2 expression groups across three cancers. **(I–Q)** Scatter plots of the correlations between CLDN18.2 expression and three immune-related genes (CTSE, CXCL17, and LYZ) in three cancers.

For upper gastrointestinal tract cancers (STAD, ESCA, and PAAD), we performed differential analysis on the high- and low-CLDN18.2 expression groups to identify genes that were differentially expressed between the two groups. The results of the volcano plot showed a large number of downregulated genes in both STAD and ESCA, but fewer genes were downregulated in PAAD. Within the upregulated fraction, only ESCA exhibited a greater number of upregulated genes, whereas STAD and PAAD demonstrated fewer upregulated genes (Figures 6A–C). Further analysis revealed that 80 of the downregulated genes overlapped and that 32 of the upregulated genes overlapped in the three cancers (Figures 6D, E). The pathway enrichment results revealed that the downregulated genes were enriched in pathways such as the downregulation of the KRAS signaling pathway, the EMT pathway, and the calcium signaling pathway (Figure 6F). The pathways enriched among the upregulated genes included the arachidonic acid metabolism pathway, the coagulation pathway, and the retinol metabolism pathway (Figure 6G).

Furthermore, various immune-related genes were identified among the downregulated and upregulated overlapping genes, and their expression profiles were analyzed between the high- and low-CLDN18.2 expression groups. The results revealed large differences in the expression of most immune-related genes between the high- and low-CLDN18.2 groups of the three cancers (Figure 6H). This finding reflects a relationship between these genes and CLDN18.2 expression in these three cancers. Moreover, for CTSE, CXCL17, and LYZ, we explored the correlation between their expression and CLDN18.2 expression in three cancers (Figures 6I–Q). The results exhibited that, with the exception of ESCA, where CXCL17 expression was less strongly correlated with CLDN18.2 expression (Figure 6M), the remaining analyses revealed strong and significant correlation. The above results indicate that in upper gastrointestinal tract cancers, high or low CLDN18.2 expression is associated with different physiological activities, particularly the activity of cancer-related pathways and the expression of immune-related genes.

### 3.6 Relationships between CLDN18.2 expression and drug sensitivity

**FIGURE 7:**
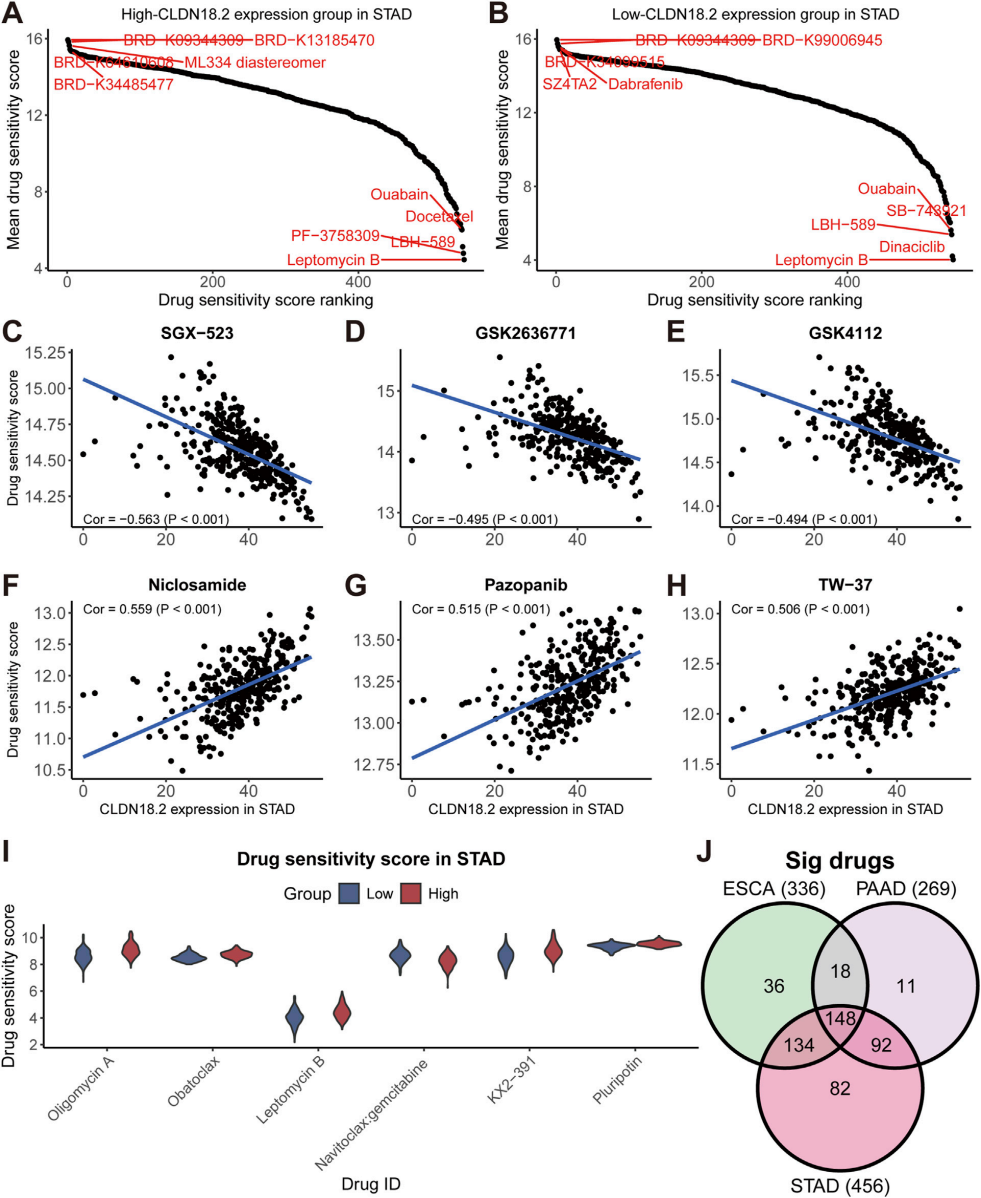
Drug sensitivity analyses in STAD. **(A, B)** Drug sensitivity score ranking diagram of high- and low-CLDN18.2 expression groups in STAD. **(C–H)** Scatter plots of the correlations between CLDN18.2 expression and the drug sensitivity score of six drugs in STAD. **(I)** Drug sensitivity scores between high- and low-CLDN18.2 expression groups in STAD. **(J)** Venn diagram of drugs with significant correlation between CLDN18.2 expression and drug sensitivity score in three cancers.

Because drug sensitivity analysis is important for cancer research, in upper gastrointestinal tract cancers, we predicted the sensitivity scores for 545 drugs and analyzed the correlation between the scores and CLDN18.2 expression (Supplementary Table S2). A higher drug sensitivity score indicated a potentially worse effect of the drug. The ranking of the drug sensitivity scores revealed that in STAD, LBH-589, leptomycin B, and quabain all had lower sensitivity scores between the high- and low-CLDN18.2 expression groups, but BRD-K09344309 had higher sensitivity scores (Figures 7A, B). Furthermore, we observed that the drug sensitivity scores of SGX-523, GSK2636771, and GSK4112 had a strongly significant and negative correlation with CLDN18.2 expression (Figures 7C–E). Conversely, the drug sensitivity scores of niclosamide, pazopanib, and TW-37 were strongly and positively correlated with CLDN18.2 expression (Figures 7F–H). We selected the top 100 drugs with the lowest drug sensitivity scores in STAD and identified six drugs that were significantly different between the high- and low-CLDN18.2 expression groups and had a correlation with CLDN18.2 expression greater than 0.4. With the exception of navitoclax:gemcitabine, the remaining five drugs were likely to have worse efficacy in the high-CLDN18.2 expression group (Figure 7I).

Moreover, we counted the overlap of drugs significantly associated with CLDN18.2 expression in the three cancers and identified 148 overlapping drugs (Figure 7J). For ESCA and PAAD, the ranking results of certain drugs, such as LBH-589, were similar to those of STAD (Supplementary Figures S5A–D). In ESCA, CLDN18.2 expression was significantly negatively correlated with the drug sensitivity scores of brefeldin A and selumetinib:MK-2206, whereas it was significantly positively correlated with the scores of PI-103 and MST-312 (Supplementary Figures S5E–H). In PAAD, the ML258 and MI-1 scores were significantly positively correlated with CLDN18.2 expression, but the effects of niclosamide and piperlongumine:MST-312 were reversed (Supplementary Figures S5I–L). Additionally, we compared the drug sensitivity scores between the high- and low-CLDN18.2 expression groups in ESCA and PAAD. The results indicated that there were more drugs with significant differences in ESCA, and selumetinib:GDC-0941 had similar effects on both cancers (Supplementary Figures S5M, N). Overall, the results of the drug sensitivity analysis reveal that several drugs may specifically treat CLDN18.2-positive tumors among these three cancers.

## 4 Discussion

This study provides a comprehensive analysis of CLDN18.2 expression and genomic alterations across multiple cancers, especially upper gastrointestinal tract cancers, and its associations with immune infiltration, prognosis, DEGs, and drug sensitivity. The results revealed that CLDN18 is expressed to some extent in various cancers, which may be related to the prevalent expression of claudin family genes in epithelial cells (Günzel and Yu, 2013). In addition, differences in CLDN18 expression between tumor samples and normal samples were present in only certain cancers, indicating that the development of other cancers may not lead to significant differences in CLDN18 expression. Further analysis of the expression of CLDN18.2 and CLDN18.1 revealed that both had strong cancer specificity and that their expression varied greatly among different cancers. CLDN18.2 is usually expressed predominantly in the stomach, but this study also revealed high CLDN18.2 expression in PAAD, CRC, ESCA, and LUAD, indicating that CLDN18.2 is ectopically expressed in these cancers. The presence of CLDN18.2 was immunohistochemically demonstrated in studies related to these several cancers, and this expression corresponded to the transcriptomic results of this study (Micke et al., 2014; Wöll et al., 2014; Moentenich et al., 2020; Iwaya et al., 2021). Additionally, unlike previous studies in which CLDN18.1 was predominantly highly expressed in lung-related cancers (Niimi et al., 2001), this study also revealed high CLDN18.1 expression in cancers such as SARC, KIRC, and KIRP. This finding shows that CLDN18.1 may also act in other tissues of the human body, which extends the research related to CLDN18.1. Interestingly, CLDN18.2 expression in cancer samples was lower than that in normal samples in STAD, which may be related to the early proliferation and invasion of gastric cancer (Nakayama et al., 2024).

In this study, the associations between CLDN18.2 expression and TMB, MSI, methylation, and CNV were investigated in ten cancers that expressed CLDN18.2. The results revealed limited associations between CLDN18.2 expression and TMB and MSI, with a positive correlation observed only in certain cancers. However, the levels of methylation and CNV differed between the high- and low-CLDN18.2 expression groups among most cancers. Moreover, analyses of the mutation profiles of STAD, CRC, ESCA, and PAAD revealed significant differences between the high- and low-CLDN18.2 groups. Among the differentially mutated genes between the two groups in STAD, certain genes have been reported to be related to gastric cancer. For example, PIK3CA mutation is associated with EBV-positive gastric adenocarcinoma (Network, 2014), and KMT2D may promote the proliferation of gastric cancer cells (Li et al., 2021). The remaining genes have been studied in other cancers (Yan et al., 2022; Song et al., 2023). These results indicate that CLDN18.2 expression is linked to genomic alterations, and the impact of genomic alterations in patients should be considered when studying CLDN18.2 in cancer.

CLDN18.2 expression was also related to the tumor microenvironment and had strong cancer specificity. For example, in STAD and ESCA, the infiltration level of NK cells was significantly lower in the high-CLDN18.2 expression group than in the low-CLDN18.2 expression group, but this difference was not observed in PAAD. This phenomenon has also been reported in a previous study related to gastric cancer (Lenz et al., 2022). Moreover, the two groups exhibited greater differences in the CD4^+^ T cell subset infiltration levels in these cancers, with smaller differences observed in the CD8^+^ T cell subsets. These results differ from those reported by Wang et al., whose study demonstrated a higher number of CD4^+^ and CD8^+^ T cells in CLDN18.2-positive gastric cancer tumors (Wang et al., 2023). Jia et al. performed a comprehensive analysis of the tumor immune microenvironment in CLDN18.2-positive gastric cancer patients. Their findings revealed no significant difference in macrophage infiltration between CLDN18.2-positive and CLDN18.2-negative groups (Jia et al., 2022). However, our study revealed a significant difference in the infiltration levels of macrophage subsets between the high- and low-CLDN18.2 expression groups in STAD, whereas no similar phenomenon was observed in ESCA, PAAD, and CRC. These results deserve further exploration.

The prognostic analysis of CLDN18.2 expression revealed that high CLDN18.2 expression was a significant risk factor in an integrated analysis of ten cancers. Specifically, in individual cancers, low CLDN18.2 expression was more conducive to prognosis in BLCA, ESAC, and PAAD. Among these cancers, CLDN18.2 appeared less common in research related to BLCA. Combined with the expression levels shown in Figure 2 for CLDN18.2 and CLDN18.1, these findings indicate that CLDN18.2 may have significance for the development of BLCA, which may expand the research in this area. Furthermore, although low CLDN18.2 expression in STAD patients predicted a better prognosis, the results were not significant. Similar situations have also been reported in several previous prognostic analyses of samples with high CLDN18.2 expression based on immunohistochemical results (Arnold et al., 2020; Kayikcioglu et al., 2023); that is, the prognosis of CLDN18.2-positive patients was similar to that of CLDN18.2-negative patients. Taken together, these findings will advance in-depth research on CLDN18.2 in the fields of immune infiltration and prognosis.

By analyzing the differences between the high- and low-CLDN18.2 expression groups in the three cancers, relevant DEGs were identified and enriched in the corresponding pathways. The downregulated pathways included several, such as the KRAS signaling pathway, the EMT pathway, and the calcium signaling pathway, all of which are strongly associated with cancer progression (Monteith et al., 2017; Oshi et al., 2024; Singhal et al., 2024). These findings indicate that high CLDN18.2 expression may affect disease progression through the disruption of intercellular signaling. The upregulated pathways included several, such as the arachidonic acid metabolism pathway, coagulation pathway, and retinol metabolism pathway, all of which are related to angiogenesis (Falanga et al., 2013; Li et al., 2017; Wang et al., 2021). This finding may indicate a connection between the upregulation of CLDN18.2 expression and the mechanism of angiogenesis in the occurrence and development of cancer. In subsequent analyses, three immune-related genes that were highly correlated with CLDN18.2 expression were identified. Among them, CTSE has been shown to synergize with docetaxel in gastric cancer research to facilitate treatment (Li et al., 2022). CXCL17 is a chemokine involved in angiogenesis and has anti-inflammatory effects (Lee et al., 2013). And LYZ is considered a potential biomarker and target for hepatocellular carcinoma (Gu et al., 2023). These findings indicate that these genes may serve as potential biomarkers to drive relevant therapies in the treatment of cancers with high CLDN18.2 expression.

Drug sensitivity analysis revealed that the effects of niclosamide were reduced in three cancers as CLDN18.2 expression increased. In addition, drugs such as dinaciclib (a cyclin-dependent kinase inhibitor), LBH-589 (a histone deacetylase inhibitor), leptomycin B (an antibiotic that inhibits the activity of nucleoplasmic transfer proteins), and quabain (a cardiac glycoside compound that inhibits the sodium–potassium pump) had sensitivity scores that were very low, indicating that they may be applicable in the treatment of multiple cancers. At present, several research advances in these drugs have been reported (Saqub et al., 2020; Shen et al., 2020; Zhu et al., 2020; Lee et al., 2021). In addition to these versatile drugs, certain specific drugs may exist for each cancer. For example, PF-3758309, also known as pictilisib, is a phosphoinositide 3-kinase inhibitor that inhibits the intracellular phosphoinositide 3-kinase pathway, which in turn inhibits the growth and spread of cancer cells. The drug is currently being used in research in combination with clofarabine for the treatment of gastric cancer (Khalafi et al., 2022). In the STAD results of our study, PF-3758309 had a low drug sensitivity score in the high-CLDN18.2 expression group, which may imply that it plays a role in the treatment of CLDN18.2-positive patients and can be further studied. Thus, our findings will likely promote relevant drug research based on CLDN18.2.

## 5 Conclusion

In summary, this study revealed CLDN18.2 expression across various cancers and its potential associations with genomic alterations, immune infiltration, and prognosis. CLDN18.2 was highly expressed in upper gastrointestinal tract cancers (STAD, ESCA, and PAAD), but high CLDN18.2 expression may be associated with poor prognosis. Combining the pathway enrichment results of these three cancers, we found that low CLDN18.2 expression was closely associated with cell signaling, whereas high CLDN18.2 expression may promote disease progression through angiogenesis mechanisms. Additionally, the immune microenvironment and the drug efficacy differed between the high- and low-CLDN18.2 expression groups, which may imply different therapeutic strategies. From a multi-omics perspective, these results indicate that CLDN18.2 has potential as a biomarker or therapeutic target in multiple cancers, especially upper gastrointestinal tract cancers.

## Data Availability

The datasets presented in this study can be found in online repositories. The names of the repository/repositories and accession number(s) can be found in the article/Supplementary Material.
