# Supplementary material for: Pan-cancer analysis of CLDN18.2 shed new insights on the targeted therapy of upper gastrointestinal tract cancers
Source: Front Pharmacol. 2024 Nov 1;15:1494131. doi: 10.3389/fphar.2024.1494131 (PMC11563798; doi:10.3389/fphar.2024.1494131)
Supplement: Supplementary file 3 [file DataSheet1.docx]

Supplementary Material

# Supplementary Figures and Tables

## Supplementary Figures


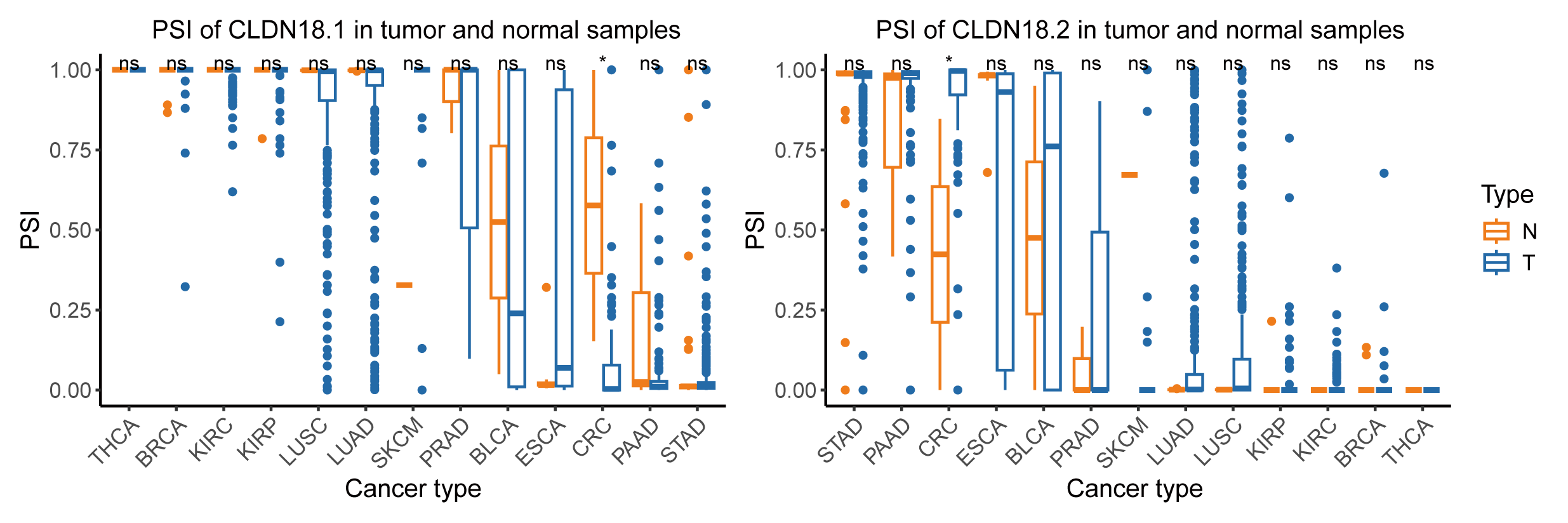


**Supplementary figure S1.** PSI values of CLDN18.2 and CLDN18.1 in normal and tumor samples across various cancers. *, P < 0.05; **, P < 0.01; ***, P < 0.001; ****, P < 0.0001; ns, not statistically significant.


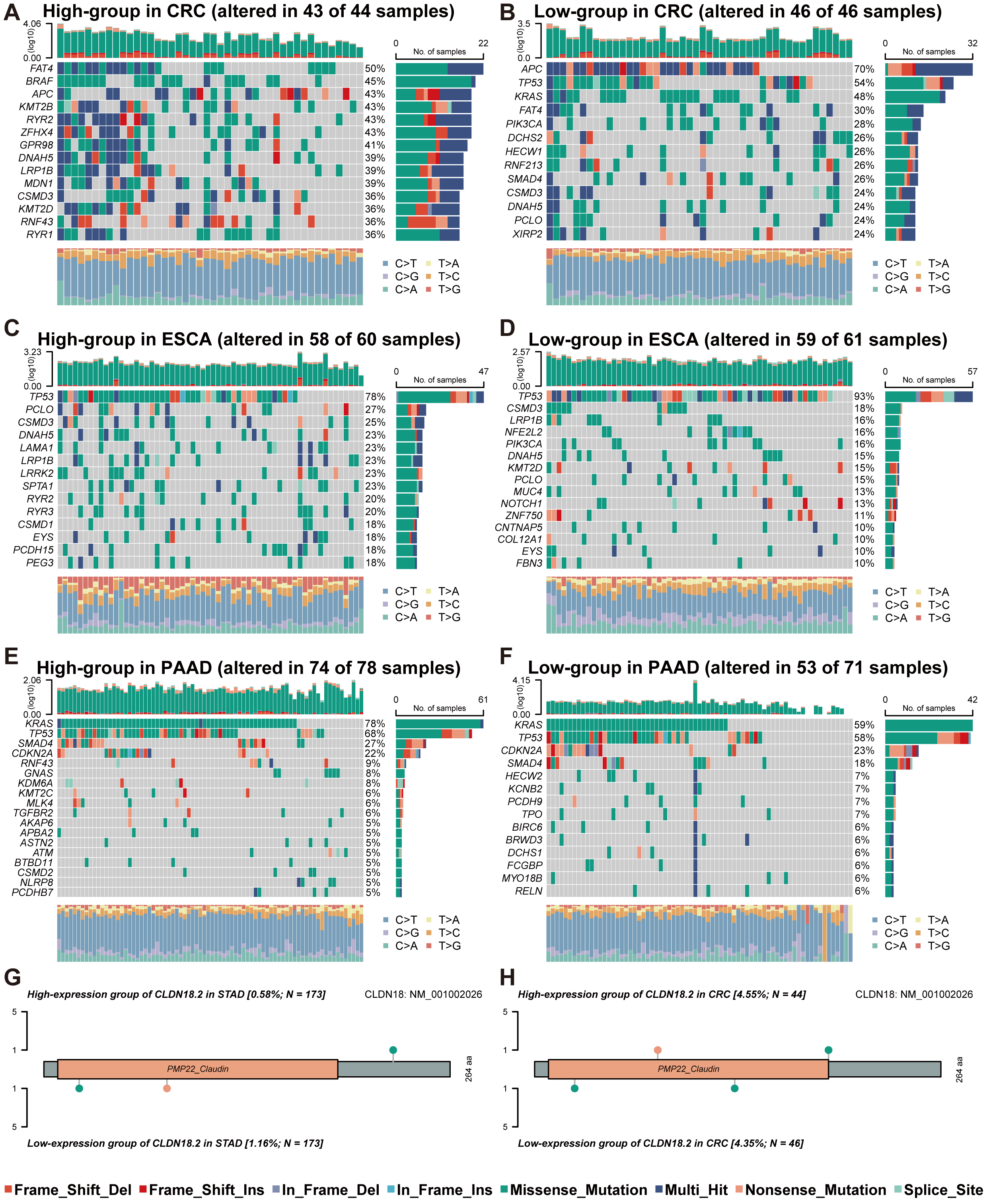


**Supplementary figure S2.** Mutation profiles in CRC, ESCA, and PAAD. (A-F) Mutation profiles between the high- and low-CLDN18.2 expression group in CRC, ESCA, and PAAD. Protein mutation lollipop plots for CLDN18.2 between high- and low-CLDN18.2 expression groups in STAD (G) and CRC (H).


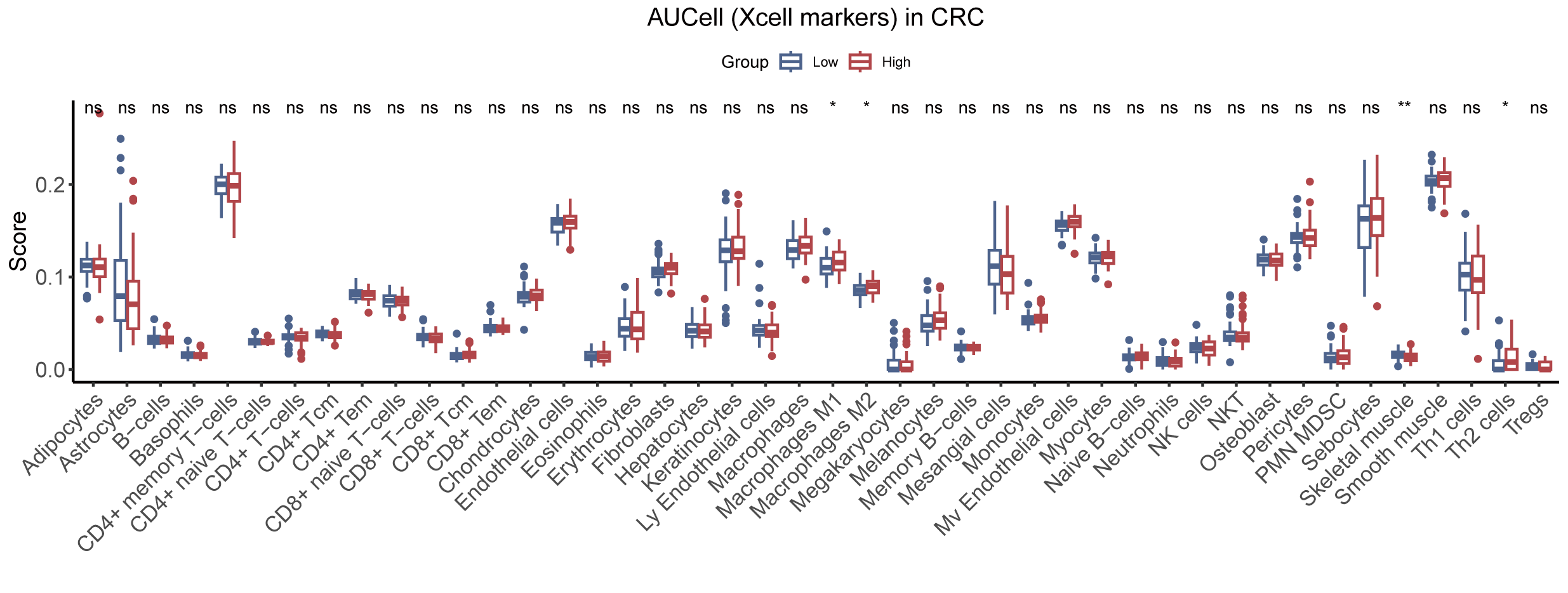


**Supplementary figure S3.** The levels of immune cell infiltration between high- and low-CLDN18.2 expression groups in CRC. *, P < 0.05; **, P < 0.01; ***, P < 0.001; ****, P < 0.0001; ns, not statistically significant.


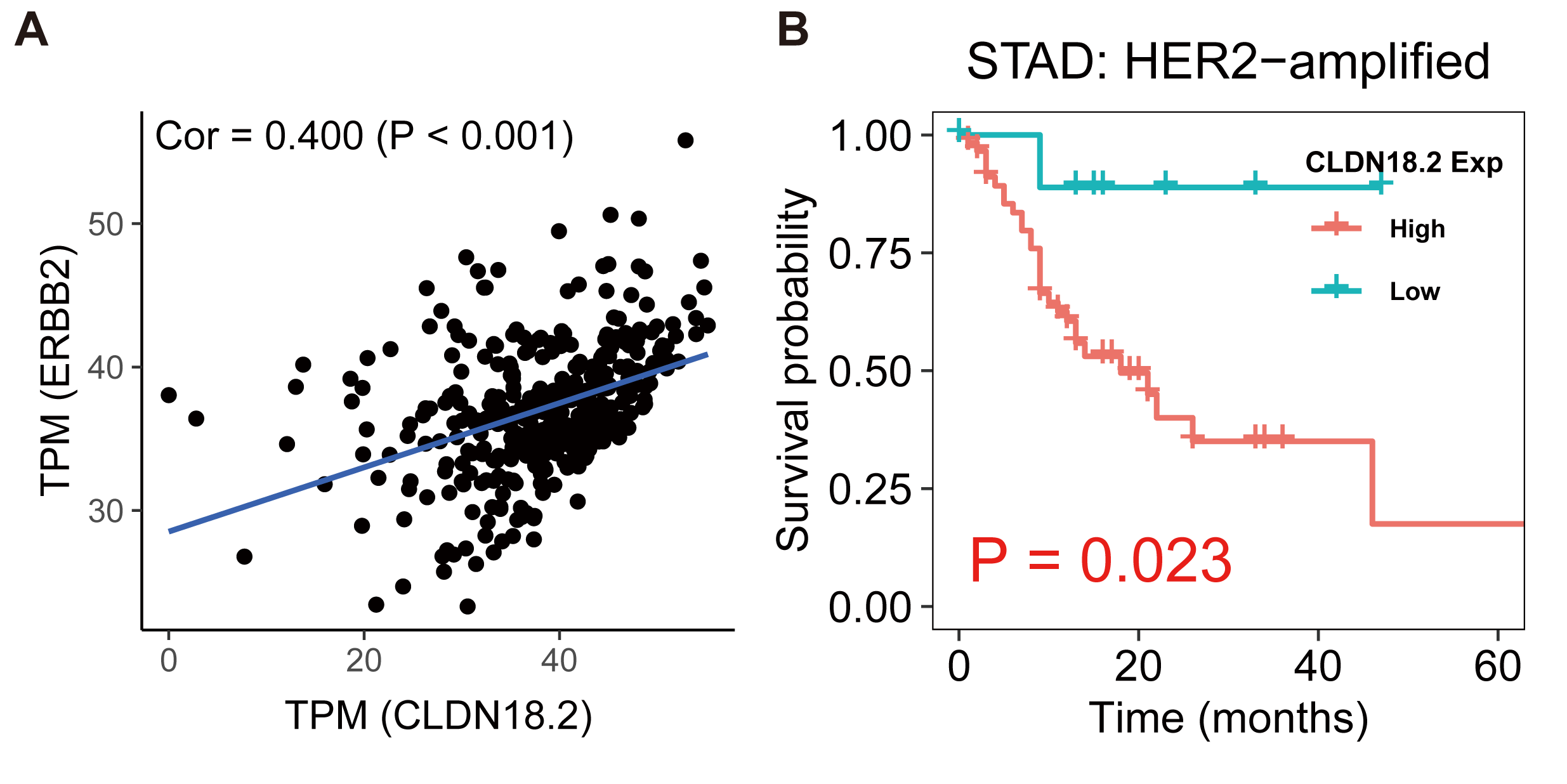


**Supplementary figure S4.** Relationships between CLDN18.2 expression and HER2 in STAD. (A) Scatter plot of the correlation between CLDN18.2 expression and ERBB2 expression. (B) Kaplan–Meier analyses of the relationship between CLDN18.2 expression and OS in HER2-amplified samples.


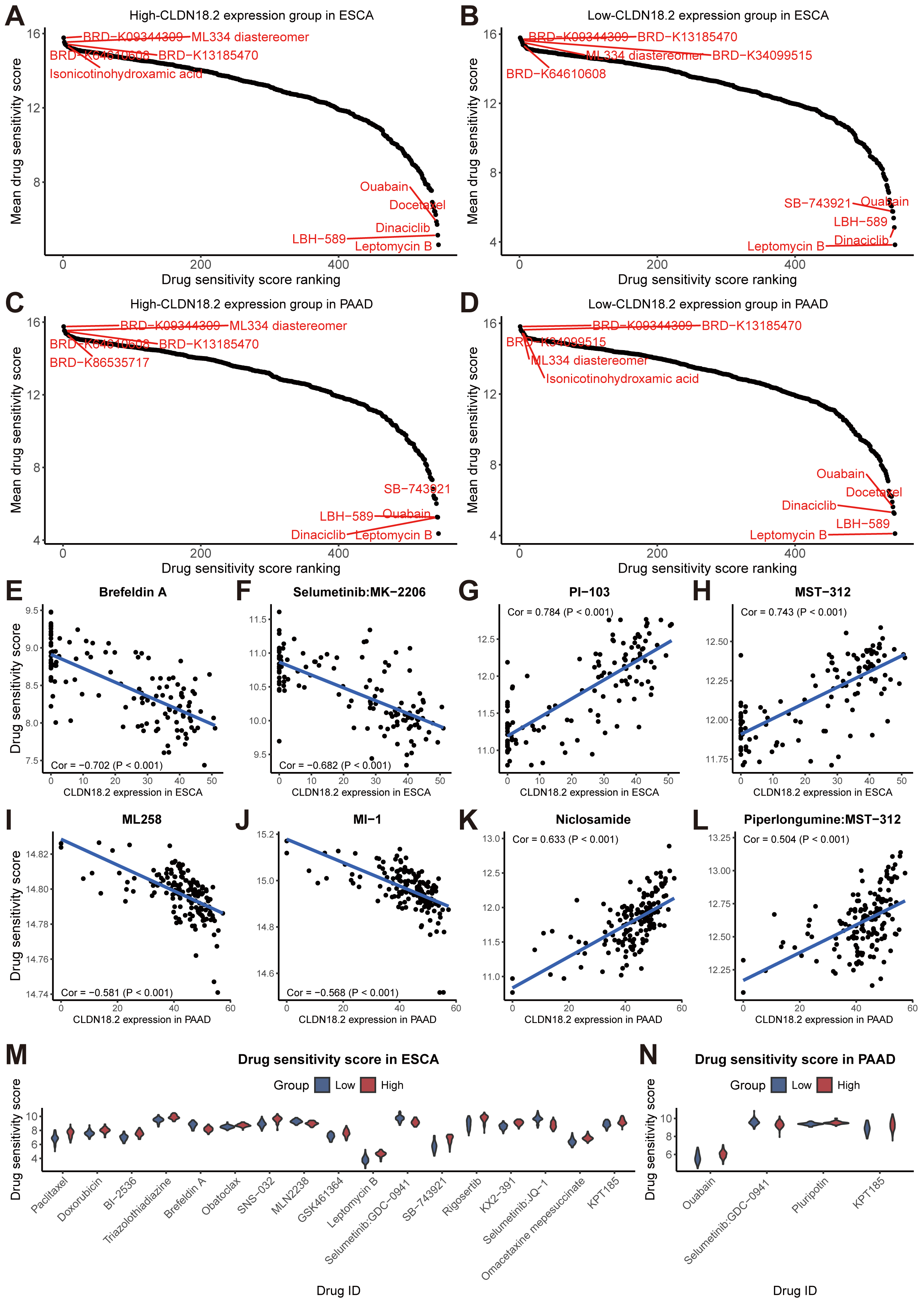


**Supplementary figure S5.** Drug sensitivity analyses in ESCA and PAAD. (A-D) Drug sensitivity score ranking diagrams between high- and low-CLDN18.2 expression groups in ESCA and PAAD. (E-H) Scatter plots of the correlation between CLDN18.2 expression and the drug sensitivity of four drugs in ESCA. (I-L) Scatter plots of the correlation between CLDN18.2 expression and the drug sensitivity of four drugs in PAAD. (M-N) Drug sensitivity scores between high- and low-CLDN18.2 expression groups in ESCA and PAAD.

## Supplementary Tables

**Supplementary table S1.** The pan-cancer data of CLDN18.2 and CLDN18.1.

**Supplementary table S2.** Relationships between CLDN18.2 expression and drug sensitivity score in STAD, ESCA, and PAAD.
